# *P. falciparum* Infection Durations and Infectiousness Are Shaped by Antigenic Variation and Innate and Adaptive Host Immunity in a Mathematical Model

**DOI:** 10.1371/journal.pone.0044950

**Published:** 2012-09-19

**Authors:** Philip Eckhoff

**Affiliations:** Intellectual Ventures, Bellevue, Washington, United States of America; Université Pierre et Marie Curie, France

## Abstract

Many questions remain about *P. falciparum* within-host dynamics, immunity, and transmission–issues that may affect public health campaign planning. These gaps in knowledge concern the distribution of durations of malaria infections, determination of peak parasitemia during acute infection, the relationships among gametocytes and immune responses and infectiousness to mosquitoes, and the effect of antigenic structure on reinfection outcomes. The present model of intra-host dynamics of *P. falciparum* implements detailed representations of parasite and immune dynamics, with structures based on minimal extrapolations from first-principles biology in its foundations. The model is designed to quickly and readily accommodate gains in mechanistic understanding and to evaluate effects of alternative biological hypothesis through *in silico* experiments. Simulations follow the parasite from the liver-stage through the detailed asexual cycle to clearance while tracking gametocyte populations. The modeled immune system includes innate inflammatory and specific antibody responses to a repertoire of antigens. The mechanistic focus provides clear explanations for the structure of the distribution of infection durations through the interaction of antigenic variation and innate and adaptive immunity. Infectiousness to mosquitoes appears to be determined not only by the density of gametocytes but also by the level of inflammatory cytokines, which harmonizes an extensive series of study results. Finally, pre-existing immunity can either decrease or increase the duration of infections upon reinfection, depending on the degree of overlap in antigenic repertoires and the strength of the pre-existing immunity.

## Introduction

Mathematical modeling of malaria necessarily includes more than population-level transmission models [1]–[5] and extends to detailed models of within-host dynamics [6]–[10]. Crucial questions about malaria infections–including those regarding the duration of patent parasitemia [11] and the extent of subpatent and asymptomatic infections in a partially-immune population [1], [10]–can be studied and explored within a suitably detailed intrahost model. These detailed models can be used to explore and to understand relationships among gametocytes, immunity, and human infectiousness to mosquitoes [12]. The local diversity of *P. falciparum* and the effects of acquired immunity on reinfection may also be important factors in elimination campaigns, and certain intrahost models can be used to explore these phenomena decoupled from either the high-dimensional complexity or the lower-resolution for infections in a full population-transmission model. Intrahost models can also serve as testbeds to examine potential vaccines and drugs early in development [4], [13]. Each species of malaria exhibits a complicated life cycle [14]–[16], and models that focus on the within-host dynamics of the parasite can provide detailed resolution of these life cycle features and their potential disruption. Finally, intrahost models can be used to evaluate hypotheses regarding parasite within-host dynamics and immune responses and to propose new experiments and field studies.

Mathematical models for studying intrahost dynamics of malaria have a rich history. Most ordinary differential equation (ODE)-based models of the human population implement constant rates of recovery from the infected compartment, which is equivalent to representing malaria infections as exponentially-distributed periods of constant infectiousness [5], [17]–[19]. More detailed models of within-host dynamics often include key biological features (e.g., discrete latencies, time-varying symptoms, and dynamic infectiousness). One type of intrahost model uses a system of ODEs to represent different combinations of immune responses as well as asexual parasite and gametocyte densities [9], [20]–[24]. ODE-based intrahost models are limited by the discrete nature of the ∼2-day parasite asexual cycle, which culminates in the release of merozoites to invade other red blood cells (RBCs), and considerable care must be taken when attempting to represent these dynamics in a continuous-time framework [25]. Some recent models built using the malariatherapy dataset [26] use a discrete-time framework to better represent underlying dynamics [6], [7], [10], [13], [27]. These detailed models can also be embedded in population transmission models [4], [28].

This paper presents a novel mechanistic intrahost model and model-based simulations of the dynamics of *P. falciparum.* The model implements a hybrid structure which couples a discrete-event simulation with full latencies for events, such as rupture of mature schizonts at an interval after merozoite invasion, with continuous-time dynamics for immune responses and parasite clearance. Parasite populations are represented by discrete counts of the number of infected red blood cells, and immune responses are represented by continuous variables. This hybrid system allows a straightforward implementation of mechanistic details of the parasite and immune response. The model is based on current understanding of parasite developmental physiology and incorporates realistic innate and adaptive host immune responses. This model follows the parasite from sporozoite inoculation through all intra-host stages to gametocyte intake in a mosquito blood meal, tracking the development of *P. falciparum* within a human host. The model incorporates current knowledge of the parasite, its intra-host targets, the human immune system, and their relevant interactions into a modular format that can readily accept new data on the underlying biology and physiology of malaria transmission and immunity.

An important application of this mechanistic model is the investigation of hypotheses for underlying mechanisms of the parasite and immune response. Mechanistic representations of basic parasite and immune processes are used to study the duration of patent parasitemia, peak parasite count, infectiousness to mosquitoes, immunological memory and reinfection, and other phenomena. Hypotheses proposed and investigated include the role of the innate inflammatory immune response in limiting gametocyte success in the mosquito, the role of evolutionary optimization of the antigenic switching rate in creating the characteristic distribution of infection durations, and the role of homologous and heterologous antigens in affecting the duration of infection on reinfection. The present model proposes and implements a powerful and flexible hybrid approach that combines discrete-event and continuous-time representations of parasite and immune mechanisms to allow a transparent investigation of mechanism impact on campaign-relevant phenomena such as duration of infections, outcome upon reinfection, and transmissibility to mosquitoes.

## Results

### Analysis of the Malariatherapy Dataset

Malariatherapy data provide much of the current understanding of the course of blood-stage *P. falciparum* in an immunologically naïve individual [26]. Infection with malaria was a therapy for neurosyphilis in the first part of the twentieth century. Treatment records for 334 patients from Milledgeville, Georgia and Columbia, South Carolina between 1941 and 1954 include infected red blood cell (IRBC) and gametocyte densities, fever, and drug treatments. Some patients were reinfected with homologous or heterologous strains, allowing study of both primary immune response and immunological memory [29], [30]. It is notable that reinfection with a strain identical to the primary infection was possible: parasite counts were reduced without realization of sterilizing immunity [29]. Malaria infections are characterized by successive peaks-in-time of asexual parasitemia. These are suppressed by immune responses, with intervals of recrudescence [31]. The maximum daily parasite count of successive peaks tends to decrease, and after approximately 5 peaks, counts may drop below the detection threshold of slide-based microscopy. The elapsed time between each of the first 5 or 6 peaks tends to average just under 3 weeks [31].

Although the malariatherapy data are an important resource, they must be used with care. Drug treatments sometimes given to reduce parasite counts rather than clear infections affected disease progression and limit some uses of the data. One approach is to select the subset of patients who did not receive drug treatment during their infection [6]. However, this strategy drastically reduces the number of cases available for analyses and also introduces strong selection biases.

Various metrics provide a sparse representation of the full dataset but limit selection bias. The first metric is the duration of measured blood-stage infection, the number of days from the first recorded parasites to the last detected parasitemia [11], [26]. When modeling elimination campaigns through the regime of sparse infections, the shape of the duration distribution becomes vital as the continuum approximation corresponding to exponential durations becomes less applicable. Sama et al. [32] showed that a Weibull or Gompertz distribution is superior to exponential for primary infections. This reanalysis includes patients who had no drug treatment within the 14 days before the last recorded parasite-positive day. Distributions are shown for all cases that were not disqualified, and then under more stringent requirements for post-clearance follow-ups at 7-, 14-, 21-, and 60-days.

**Figure 1:**
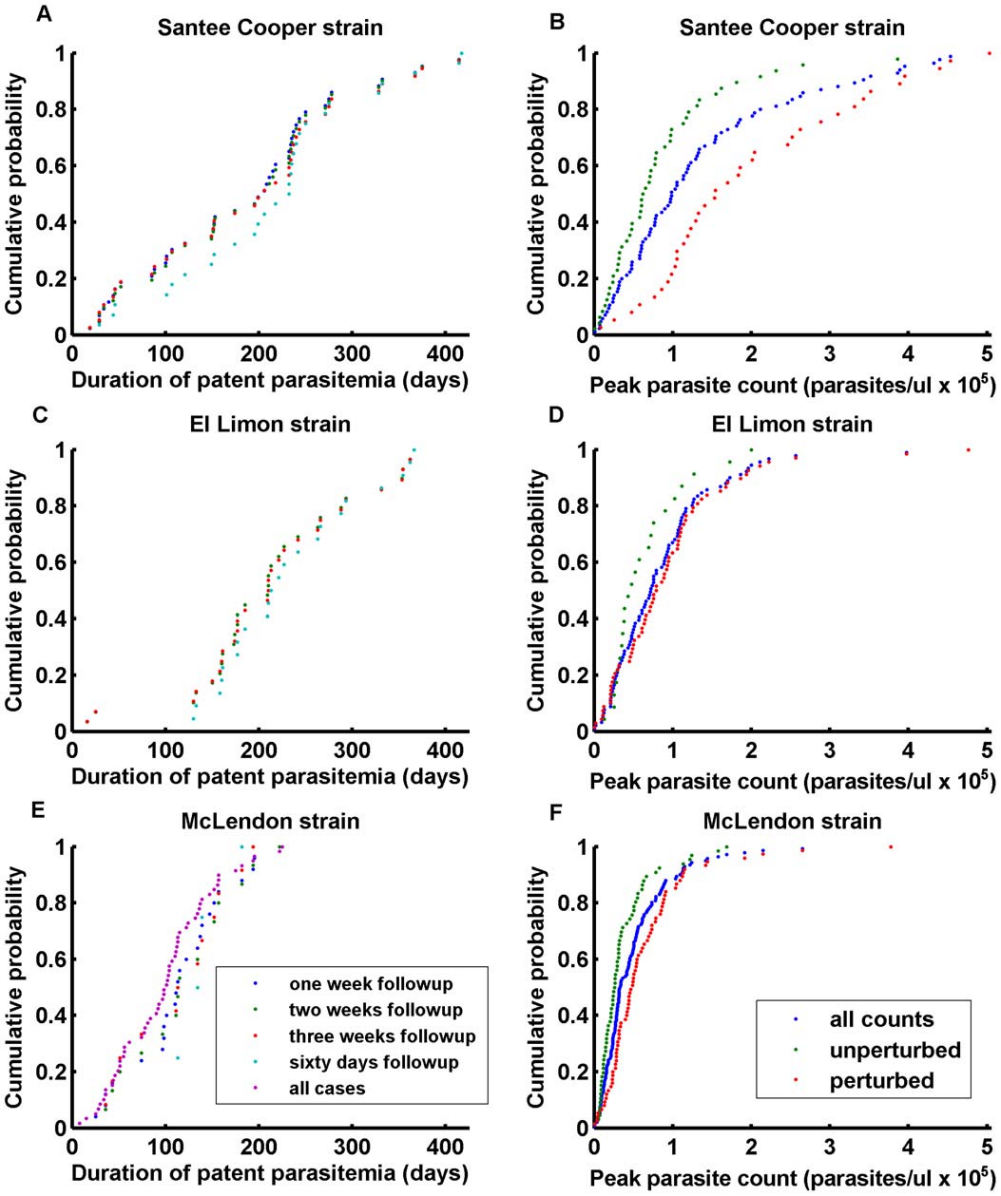
Measured durations and peak parasite counts in the malariatherapy data. Durations (A,C,E) and peak parasite counts (B,D,F) from malariatherapy dataset for the Santee Cooper, El Limon, and McLendon strains. Inclusion criteria are described for durations and peak parasite counts. For the latter, “unperturbed” implies no drug treatment before the fifth day after the measured peak. Even perturbed cases (light blue) are included, since these cases requiring drug treatment represent possible high-parasitemia outcomes.

For each strain, the distribution of duration shows an interval of high probability density about the mean, with a tail in each direction (Figure 1 A,C,E). Mean durations differ, with an average (given a 60-day follow-up) of 216 days for Santee Cooper, 233 for El Limon, and 142 for McLendon. The McLendon mean derives from only 4 cases with a 60-day follow-up; dropping the required follow-up period to 21 days reduces the mean duration to 115 days.

Peak parasite count is another important metric. Individual cases are classified by strain and drug treatment. Cases with drug treatments <5 days after the recorded peak parasite count are classified as perturbed. These cases have a higher mean for all 3 strains. This is partly the result of high parasite counts and associated fevers requiring more aggressive treatment for patient safety. All cases, rather than just the unperturbed ones, are used to constrain model results. This ensures that the perturbed high-parasite count cases inform possible outcomes (Figure 1 B,D,F).

### Strength of Immune Response

**Figure 2:**
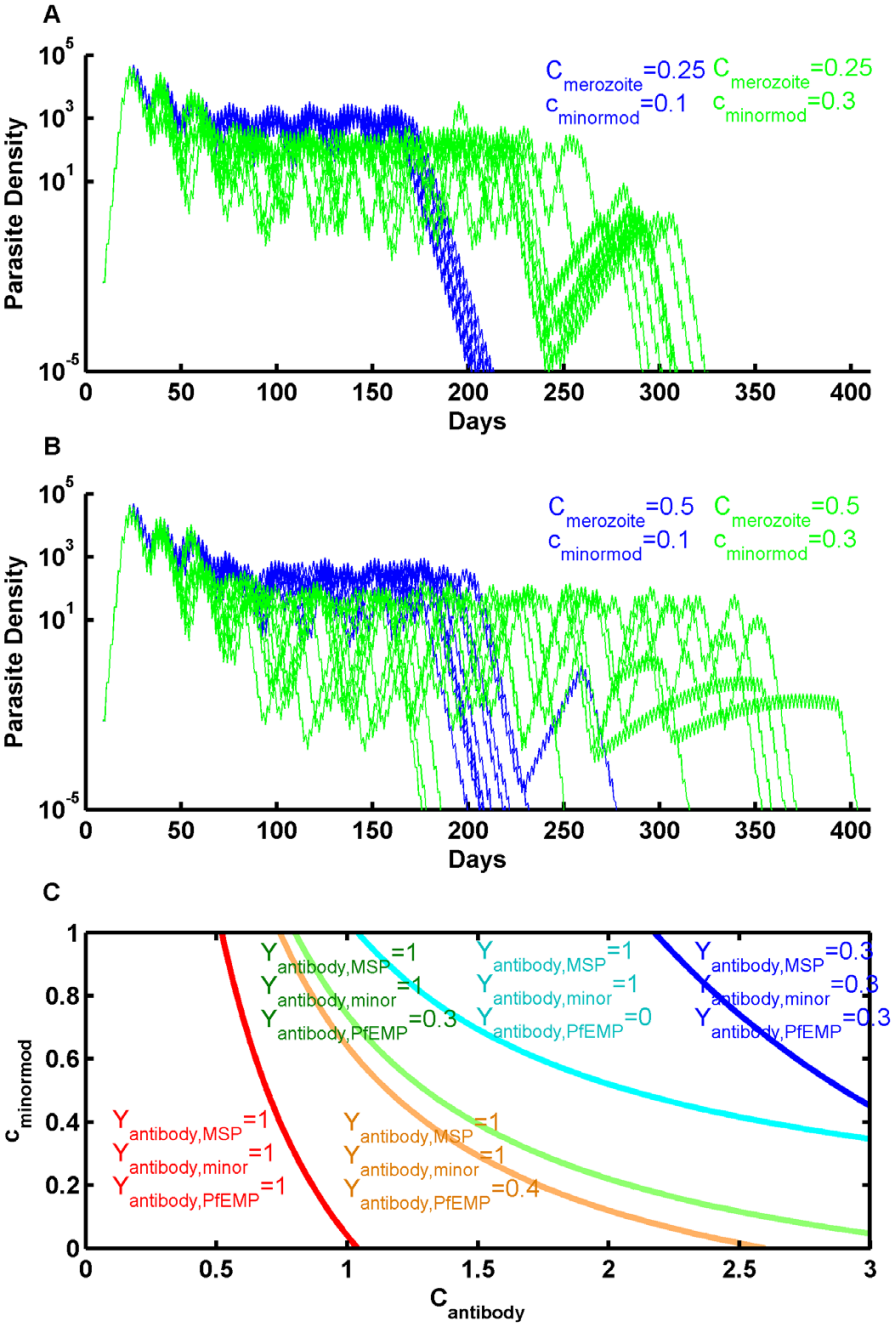
Effect of varying non-specific antigenicity c_minormod_ and antibody kill rate C_antibody_ on immune-parasite interactions. A,B) Simulated infection trajectories for different sets of immune parameters, for *C_antibody_* = 1.5. Increased shared immunity reduces parasite densities, extends the duration of the first pass through the antigenic repertoire, and increases trajectory variability. C) Each curve corresponds to a level set of immune response which over a two-day cycle balances the merozoites per schizont. The red curve corresponds to maximum antibody levels, exhibited for Y_antibody,MSP_ = 1 for merozoite-specific immunity and Y_antibody,i = _1 for the PfEMP-1 minor epitope-specific antigens. To the left of the red curve, no clearance occurs for any variant of the infection. The orange curve corresponds to Y_antibody,MSP_ = 1, Y_antibody,i = _1 for minor epitope-specific antibodies, and 0.4 for PfEMP-1. The green curve is the same as before, but with Y_antibody,i_ = 0.3 for PfEMP-1 specific immunity, which is the level at which antibody is first released. The cyan curve is for Y_antibody,i_ = 0 for the expressed PfEMP-1 variant. To the right of this curve, new variants cannot grow in the presence of maximum non-specific antibody responses to merozoites and minor epitopes. Finally, the dark blue curve corresponds to Y_antibody,i_ = 0.3 for all three antibody responses. These curves can be used to constrain realistic parameter ranges.

Model features and parameters influence infection severity, duration, infectiousness, or the pattern of recrudescence during long infections, and parameter studies can explore the effects of different components of immunity and the potential of vaccines enhancing specific responses. Figure 2 illustrates the relationships among merozoite-blocking immunity ***C_merozoite_***, antibody clearance of *P. falciparum* erythrocyte membrane protein 1(PfEMP-1) major variants ***C_antibody_***, and antibody responses to the shared minor epitopes ***c_minormod_***. Every 2 days, IRBC schizonts rupture and release an average of 16 merozoites per schizont. If the merozoite-blocking antibody response (characterized by ***Y_capacity,MSP_*** and ***Y_antibody,MSP_*** ) is at full strength, then ***C_merozoite_*** determines the number of new IRBCs. Each will express a PfEMP-1 variant and a minor epitope as in [33]. The antibody response to these two antigens influences how many IRBCs survive to rupture.

Figure 2 A,B show ten simulated infection trajectories for different combinations of immunity parameters. Before the parasite density rises to a level inciting an innate response, there is very little parasite clearance. Increasing shared adapted immune responses, either merozoite-blocking immunity, or immunity to shared minor epitopes reduces the overall parasite density envelope as immunity develops and extends the duration of passage through the antigenic repertoire, indicated by the major drop in parasite density. Extending much beyond 200 days allows reuse of antigenic variants and recrudescence with reduced parasite density. Note that the lower shared immunity results in higher parasite densities and a more deterministic infection, but that reduced parasite densities and extended durations result in much more infection variation, even with fixed parameters. The highest shared immunity corresponds to the broadest distribution of infection.

Each curve in panel c) corresponds to a set of fixed antibody levels for merozoite inhibition ***C_merozoite_*** = 0.5. They divide the plot area into values for kill rates that diminish the parasite population below the replacement level allowed by merozoite immunity (right and upwards of each curve) and those for which the parasite will continue to proliferate until antibody levels increase (left and downward). The x-axis shows the value of the antibody kill rate ***C_antibody_***; the y-axis, the value of non-specific antigenicity ***c_minormod_***. Known characteristics of infection dynamics place firm constraints on the covariation of model parameters, thereby reducing the effective degrees of freedom. Higher values of ***C_merozoite_*** shift each curve left, which may also be the effect of potential vaccines that reduce the success probability of merozoite invasion [34].

### Duration of Infections

Malaria campaign planning is impacted not only by the mean duration of infections, but by the distribution of durations, especially any long-duration tail. Important work has been done to understand the distribution of infection durations [11], [32], and mechanistic models can explore which immune and parasite mechanisms create these distributions and how the distribution changes with respect to their parameters. The model reproduces empirical duration distributions entirely through the mechanistic implementation of antigenic variation and immune responses. The high probability-density region near the mean corresponds to the time for the infecting pathogen to exhaust all available variants in its antigenic repertoire. The tails extending from this region correspond to early clearances in which the infection is cleared without exhausting its antigenic repertoire and to long duration infections which use the repertoire more than once. Clustering observed in the epoch of these early clearances has a simple explanation. Infections are characterized by successive peaks of parasitemia that introduce new antigenic variants before they are suppressed. Clearances occur in troughs, and observed correlations in peak times create correlated troughs.

A blood concentration of 200 parasites/µl corresponds to ∼10^9^ total parasites in an adult human, and for early parasite clearances to be possible, the probability of an IRBC expressing a novel variant and not the prior one needs to be close to that reciprocal. This could be the product of a high on-switch rate and low off-switch rate or vice versa [27], but it needs to be ≤10^−9^ or no early clearances occur. Higher switch rates are possible for either the on or off rate [27], [35]. In the model, ***K_antigen_*** is the overall probability of a singly-expressed variant switching to another singly-expressed variant.

**Figure 3:**
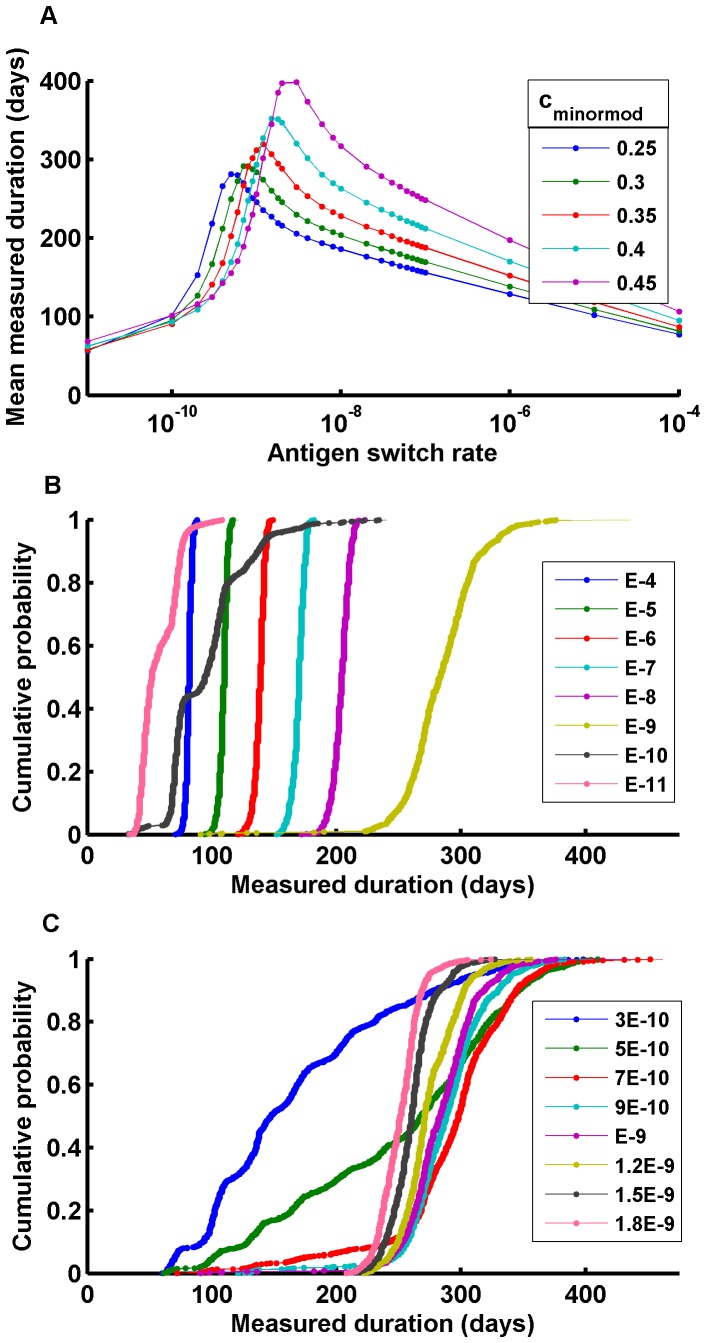
Measured infection durations governed by host immune reponse and parasite antigenic switching. A) Effect of antigenic switch rate on mean infection duration for five values of non-specific antigenicity c_minormod_ (X_50,innate = _1000, X_50,antibody_ = 10 with min stimulation 1/100, C_antibody_ = 1.5, nonspec_growth = 0.5, C_innate_ = 0.2, merozoite growth 0.05, merozoite antibody invasion blocking 0.5); B) Duration distributions for a range of antigenic switch rates K_antigen_ with c_minormod_ = 0.3; C) Duration distributions for antigenic switch rates near maximum duration have the characteristic distribution shape for *P. falciparum*.

Figure 3A presents a series of curves showing mean measured duration over a range of switch rates for different values of ***c_minormod_***, the immune response to shared minor epitopes. At sufficiently fast switch rates, soon all available antigens have been expressed, have evoked a response and have been cleared. At sufficiently low switch rates, new antigens do not appear fast enough, and clearance occurs; in an intermediate range, however, longer infection durations appear. Lower parasite densities reduce the effective variant introduction rate, slowing parasite and immune dynamics, so maximum infective durations increase with nonspecific immunity. Slower switching exhausts variants at a slower rate, extending the duration of infection until further slowing fails to outrace adaptive immunity. Although the parameters are different, the qualitative feature of an optimal switch rate is preserved, and it is thus interesting to observe the duration distributions near this peak, as parasites should have been selected for optimizing transmission.

Figures 3B,C show duration distributions from the ***c_minormod = _***0.3 curve in Figure 3A. These change from being very narrow to more closely resembling curves from the malariatherapy data as the switch rate approaches values that maximize infective durations. In Figure 3B, for fast switching, the distributions are narrow and increase in their mean as the switch rate decreases. As the switch rate slows past 10^−9^, the mean begins to drop again and the distribution widens. Two longer tails become visible, one extending to shorter durations, corresponding to clearance before exhaustion of available variants, and the other to longer durations. As the switch rate continues to fall, distributions are composed of mostly early clearances. The full transition can be seen in Figure 3C. The general shape of curves is robust to parameter variation, and these characteristically-shaped curves appear when the switching rate is slightly slower than that which produces the maximum expected duration. It is feasible to specify parameter sets that closely match each of the three strains by changing the number of variants in the repertoire, variants available to switch, and the switch rate per available variant.

The memory level for the adaptive immune response has its strongest effects on re-infection, but it also changes the long-duration tail of the distribution for initial infections. One of the key results of this model concerns the motivations for the parasite to maintain a sufficiently large repertoire of antigenic variants and to use them sparingly. Immune responses to earlier antigens wane over long intervals without their expression. Not only does this allow for future reinfection, but if the original variants are no longer suppressed and are allowed to be expressed after a long interval, another passage through the parasite’s repertoire is possible.

**Figure 4:**
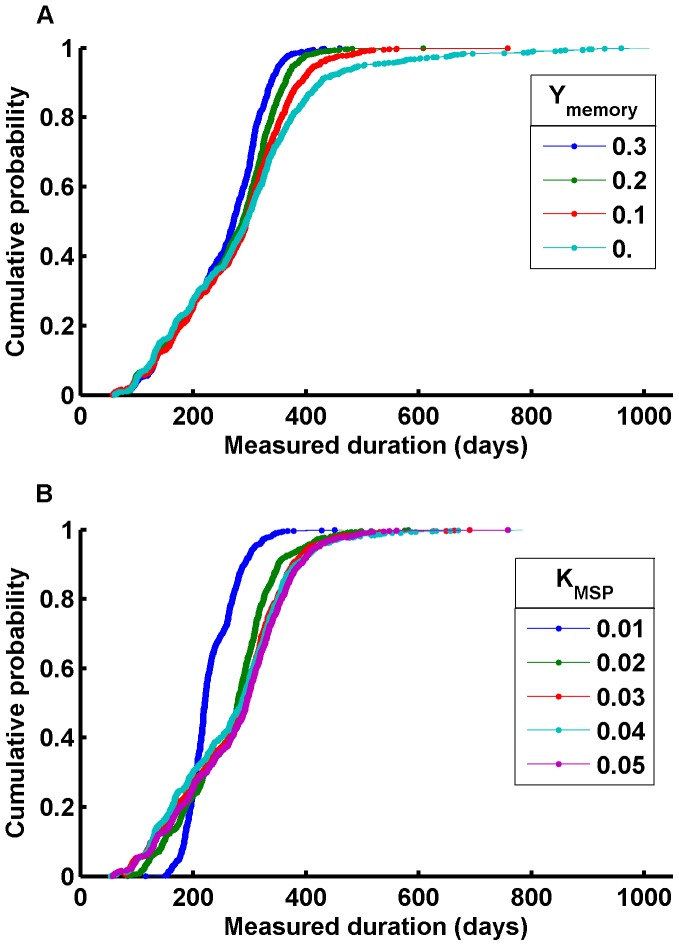
Effects of immunological memory and merozoite invasion-blocking immunity. A) Effect of memory level Y_memory_ on duration distribution; B) Effect of merozoite-specific antibody capacity growth rate K_MSP_ on duration distribution. Infection parameters are the same as they are in Figure 3, but K_MSP_ is fixed at 0.05 in a), and Y_memory_ is fixed at 0.1 in b).

One may argue that the size of the antigenic repertoire is driven to allow durations beyond the durability of the human hyperimmune response, allowing reruns utilizing the same repertoire. This “another time around” is quite different from the first pass, since now present memory B cells generate swifter antibody responses. As ***Y_memory_*** tends towards zero, the tail of the distribution extends (Figure 4A) although all infections are eventually cleared, with clearances each time through the repertoire. The interval from one high transmission season to the next also drives repertoire size, as humans provide a more reliable low-season reservoir than mosquitoes. In much of the world, a 200–240 day mean duration suffices.

The duration distribution is also influenced by the rate of development of merozoite-blocking immunity ***K_MSP_*** (Figure 4B). Slow development maintains higher parasitemia, which limits the number of early clearances and speeds the progression through the antigenic variants for a given switch rate. As merozoite-specific immunity develops more rapidly, parasitemia is suppressed, increasing the probability of early clearances, slowing exhaustion of the antigenic repertoire, and extending the durations of infections for a given switching rate.

### Peak Parasitemia

Simulations can be used to study the peak parasite count experienced during an infection and the effect of heterogeneity in individual immune responses. Stochastic simulation of the model with fixed parameters gives the desired broad distributions for duration, but the first wave of parasitemia is more deterministic due to the short interval and the large number of parasites. Thus, fixed parameters in the present model don’t capture the distribution of peak parasite counts (Figure 1 B,D,F), almost all of which occur during the first wave of parasitemia.

**Figure 5:**
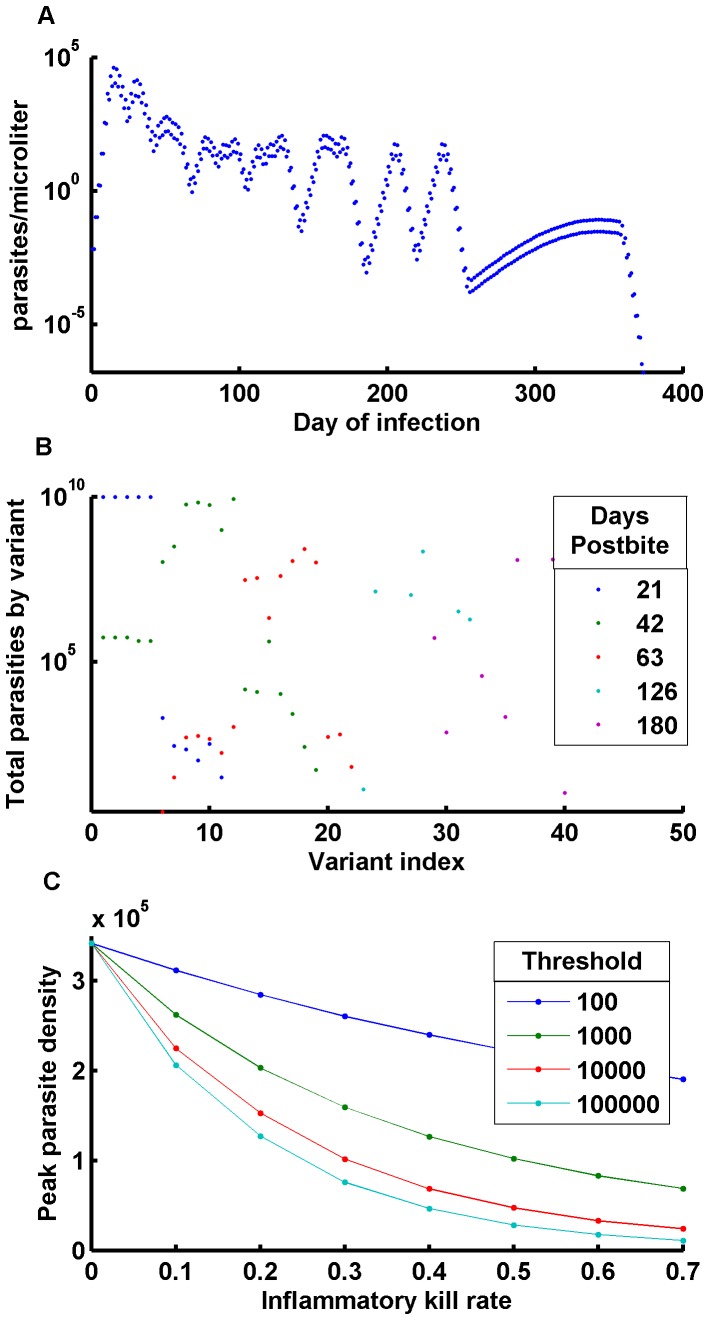
Sample infection with antigenic variation and the effect of inflammatory response. A) Sample infection with daily reports of IRBC/µl; B) Change in the distribution of expressed variants over the course of infection;. C) Effect of innate immune effective threshold X_50,innate_ and inflammatory kill rate C_innate_ on peak parasite count.

It’s possible to capture this variation with modifications of parameters intrinsic to the parasite (e.g., the asexual cycle reproductive rate per variant), or of parameters intrinsic to the immune response (e.g., thresholds), or both [6], [7], [13]. This variation is modeled with heterogeneity in individual immune responses, particularly in the inflammatory innate immune response, the variation thereof being well-known, and variations in ***C_innate_*** and ***X_50,innate_*** do modify the peak parasite count (Figure 5C). Varying a single one of these parameters is enough to match the distribution, with a fixed threshold and a uniformly distributed kill rate performing best. For fixed ***C_innate_*** and varying ***X_50,innate_*** to match the data, the threshold must vary over many orders of magnitude. If the variations in observed peak parasitemia are explained through heterogeneity in ***C_innate_***, the implication is that higher parasitemia will be observed in individuals for whom the innate immune response is weaker at clearing parasites or limiting growth.

Simulated infections recreate the characteristic series of parasite peaks with a spacing of 16–21 days, with a gradual descent in parasite peaks (Figure 5A). The simulation tracks actual parasite levels, even below detection thresholds, and exhibits a realistic pattern of recrudescence. Examining the distribution of expressed variants through early, middle, and late-infection in a simulated infection shows an initial high count of a limited number of variants, then a bloom of variants at intermediate densities. These are followed by scattered variants at low densities (Figure 5B). Other parameters, such as the merozoites per hepatic schizont ***N_livermerozoites,_*** have minimal effect on durations and peak Parasitemia for low numbers of hepatic schizonts. The number of initially expressed variants can affect peak Parasitemia, and a very high number of initial schizonts can affect acute phase dynamics as well. If the number of initially expressed variants depends on the number of liver schizonts, that would provide a possible mechanism for protection against severe outcomes by a pre-erythrocytic vaccine limiting sporozoite success. The effect of the number of initially expressed variants is stronger for a weaker innate response.

The time between successive parasite peaks is influenced by a variety of factors. Higher switch rates seed the wave of variants with more parasites expressing new variants. This results in fewer required multiplication cycles to get to the next peak. Merozoite-blocking immunity and immunity to shared minor epitopes suppress parasite populations, slow both their growth and the resulting development of antibody defenses to new variants, and extend the interval between peaks.

### Infectiousness

Human infectiousness to blood-feeding mosquitoes informs population-level models of malaria. It is difficult to develop a simple relationship between gametocyte densities and infectivity at higher gametocyte counts [12], [36]. Previous models sidestep this difficulty either by fitting time-lagged asexual parasitemias [37], assigning discrete values of infectivity based on symptomatic state [1], or fitting a sigmoidal curve through the data. Current research focuses on the characteristics and dynamics of transmission-blocking immunity, its age-dependence, and the role of the adapted response.

**Figure 6:**
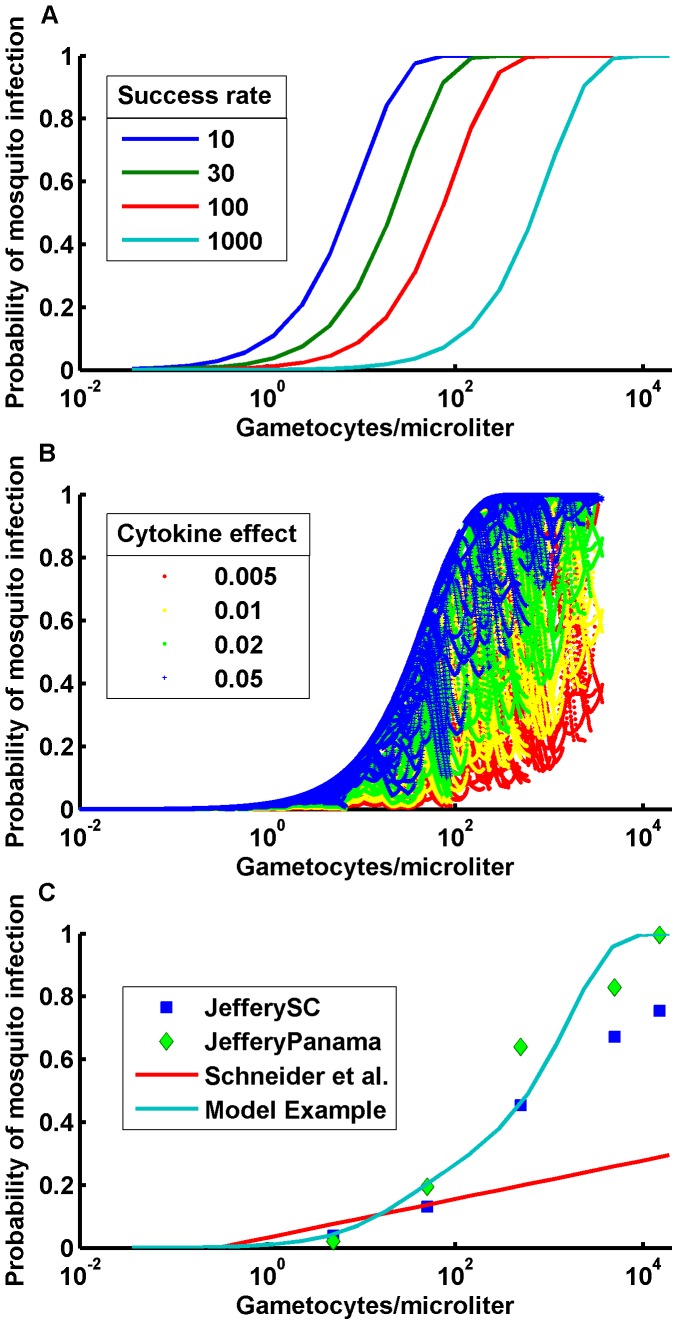
Infectivity to mosquitoes is governed by gametocyte density and other factors. The probability of mosquito infection versus gametocyte count for A) varying survival factors; B) varying effect of inflammatory immune response on gametocyte success. Inflammatory cytokine mediated gametocyte inactivation can explain widely varying mosquito infection rates even for a single individual; and C) data for the probability of mosquito infection with a model example survival factor of 100 and a cytokine effect of 0.94.

The model contains a raw count of mature gametocytes with explicit dynamics for production and decay. The number of female gametocytes per mosquito bloodmeal is then multiplied by a success factor and an inflammatory immune factor. Figure 6A shows the effect of varying the success factor with no inflammatory immune effects. Changing the success factor per female gametocyte merely shifts the sigmoid on a log scale (Figure 6A), and the sigmoid transition is far steeper than the averages in the Jeffery and Eyles data [38] (Figure 6C). Adding in the probability of male gametocytes can be done explicitly or implicitly as one of the factors that reduces the success of female gametocytes. When the success factor depends on male gametocyte density, it does not affect the high gametocyte density part of the infectivity curve where male and female gametocytes are plentiful, but reduces the low density success rate. This makes the transition from low to high infectivity even sharper, or opposite that observed in the mosquito-feeding infectivity data.

Mechanistic models can provide a testbed for hypotheses about the effect of different components of the immune response upon transmission. Adapted immunity reducing the asexual parasitemia will indirectly reduce gametocyte densities by reducing their source term. One proposed direct immune mechanism would be naturally-acquired transmission-blocking immunity based on gametocyte-specific antibody production. Such adapted responses would develop over time and exposure to antigens. As a result, however, these gametocyte-specific responses will occur at lower gametocyte counts driven by suppressed asexual parasitemia, to which adapted responses have also developed. As a result, adapted immunity to gametocyte antigens will tend to reduce the lo-gametocyte density part of the infectivity curve rather than its high-density section, and will tend to rotate the sigmoid the wrong direction. Such concerns would not apply to a transmission-blocking vaccine, which could reduce transmission over broad density ranges.

This discrepancy suggests that some additional factor decreases the success per female gametocyte at higher densities. Thus, it is reasonable to examine potential immune responses which are stronger at higher gametocyte densities. Gametocytes are produced by a subset of IRBC’s and thus, higher gametocyte densities tend to follow higher asexual densities. Higher asexual densities, especially those that occur while a specific adapted immune response is still low, are often correlated with high levels of inflammatory cytokines. Experiments with crisis serum transfer show transmission-blocking immunity [39] due to cytokine-mediated inactivation [40], confirming a potential role for the innate response in limiting transmission. Such a role is indeed suggested by model simulations and harmonizes a series of studies.

An inactivation factor based on the level of inflammatory cytokines decreases the effectiveness of gametocytes in the calculation of infectivity to the mosquito. Varying the strength of this effect shapes the infectivity curve appropriately (Figure 6B). Individual heterogeneity in the innate immune response would then drive some heterogeneity in infectivity, but even for a fixed set of parameters, there is a broad scatter in model outputs for gametocyte densities versus the inactivation factor which increases with the factor (Figure 6B). A given gametocyte density does not map to a single cytokine level; the relationship depends on the stage of the current infection and antigenic history prior to a given infection. As inflammatory cytokine levels decrease for a given asexual parasite density, the infectivity for a given gametocyte density approaches the original unperturbed sigmoid in Figure 6A. In the present model, immune response thus limits transmission in two ways: by suppressing asexual parasitemia on reinfection, thereby lowering gametocyte density; and innate immune responses limiting the overall transmission effectiveness per gametocyte.

### Reinfection

A final set of questions which can be examined through a mechanistic model focus on the effects of pre-existing immunity on new infections. Reinfection with homologous or heterologous strains has been previously described empirically by Jeffery and Collins [29]. Immunity to severe malaria is seen after as few as 1–2 infections [41], but it can take much longer to attain effective parasite immunity [1], [4], [5]. Malaria infections do not result in sterilizing immunity (patients were reinfected with an identical strain after clearing their primary infection [29]). Peak parasite counts were less than those in the primary infection, showing some immunological memory even years after the initial infection. Compared to the primary infection, fever was lower and IRBC and gametocyte densities tended to be reduced [29]. It is useful to study *in silico* how durations, severity, and transmission may vary on reinfection.

In addition to the antigenic variation of expressed PfEMP-1 variants during a single infection, different clones of *P. falciparum* can have different repertoires of available variants [42]. The total number of available variants in a given geographical area, let alone in the world, is unknown, but similar variants have been found in distant areas [43]. The greater the number of variants, the longer it takes to build up the array of protective, but not sterilizing, immune response, and ‘holes’ in the antibody repertoire exist even after years of exposure. The present model with specific antibody responses to an array of possible antigens allows study of the effect of parasite diversity upon the acquisition of protective immunity through varying the parasite antigenic repertoire upon reinfection.

The outcomes of repeated reinfection depend on the degree of overlap of the infection’s antigenic repertoire with prior experienced antigens. Antigenic variation has also been observed for the protein AMA-1 in merozoites, which has been observed to be an immune target [44]. Several variants for the merozoite antigen type are included in the model (with MSP-1 in the model representing the collection of immune targets on merozoites), and different available sets of minor epitopes. Each infection in the model is characterized by a single merozoite antigen type, a single set of minor epitopes, and 50 PfEMP-1 variants. If there are m merozoite antigen types and n sets of minor epitopes, then there are m*n possible combinations of non-specific antigenic profiles for infections, with different degrees of cross-immunity due to shared antigens.

**Figure 7:**
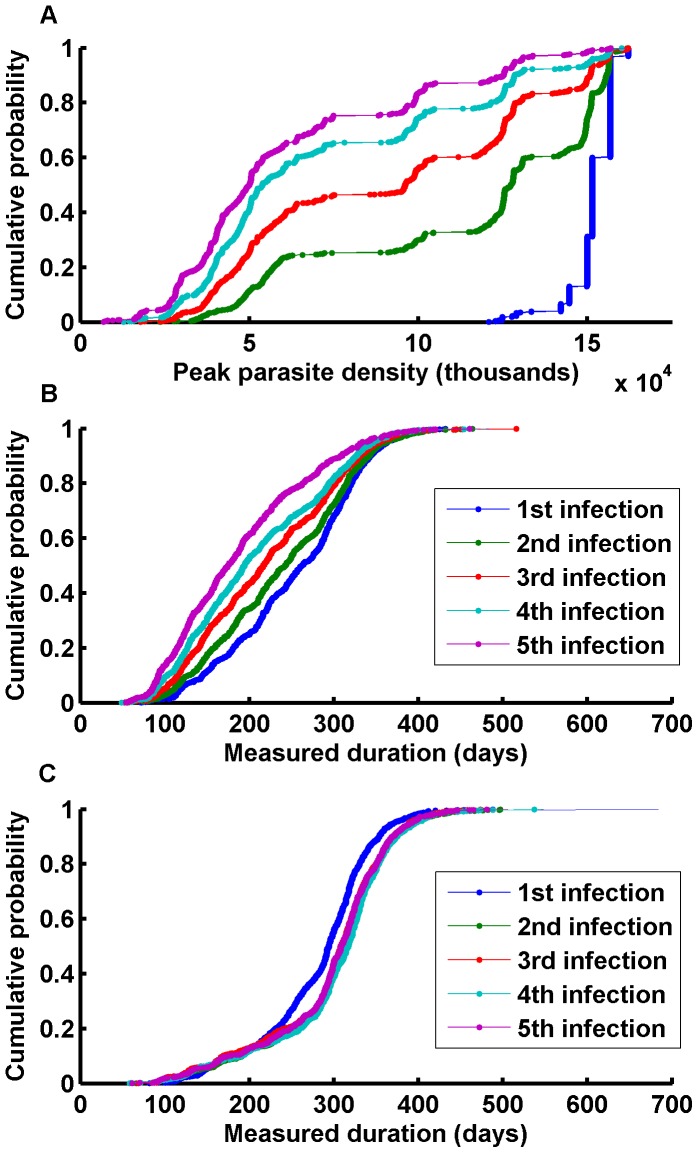
Measured durations and peak parasite counts for reinfection. Reinfection studies for the changes in A) peak parasite density; B) measured duration for random antigen selection; and C) measured duration for fixed minor epitope set and MSP variant but disjoint PfEMP-1 sets. The distributions of peak parasite count and measured duration are compared for the 1^st^–5^th^ infection. Effect of reinfection on duration depends on the antigenic overlap between primary and secondary infections.

The present simulations implement a strain population with 300 possible PfEMP-1 variants, 4 MSP-1 variants, and 4 sets of 5 minor epitopes. In the first case, individuals are infected with a series of strains with a 300-day interval from clearance to reinfection to allow immunity to return to ***Y_memory_***. Each strain has a set of 50 PfEMP-1 antigens drawn at random from the 300, 1 MSP-1 variant, and 1 set of 5 minor epitopes. Each of the 50 PfEMP-1 variants receives 1 of the 5 minor epitopes at random. As an individual develops a broader immunological repertoire, fewer cases have high peak parasite counts. Infections are shorted by pre-existing immunity, but the effect on peak parasitemia is much more dramatic than that on duration (Figure 7 A,B).

Durations are prolonged when the MSP-1 variant and set of nonspecific antigens are maintained, but each set of PfEMP-1 variants is entirely novel (Figure 7C). Although pre-existing immunity to MSP-1 and minor epitopes limit parasitemia, it’s not enough for clearance of novel PfEMP-1 variants. Lower parasite counts slow progression through available variants and result in longer durations. Whether immunity speeds or slows clearance has been the subject of extensive debate, and investigating fundamental immune and parasitological mechanisms in the context of this model illuminates the conditions under which each effect would be observed.

The earlier analysis maximized average duration of infection for the primary infection, but reinfection also affects optimal antigenic switch rate. During a secondary infection with homologous antigens, a higher switch rate allows variants to be introduced at lower parasite densities. This is typical of secondary infections with previously experienced antigens. Thus, the optimal switch rate for secondary infections is higher. In moderate to high transmission areas, where most infections are secondary, an evolutionarily-driven switch rate should be higher. Conversely, it should be lower in low-transmission areas, where primary infections make up most cases. It is worth noting that most of the malariatherapy strains were from low- to moderate-transmission regions [26].

**Figure 8:**
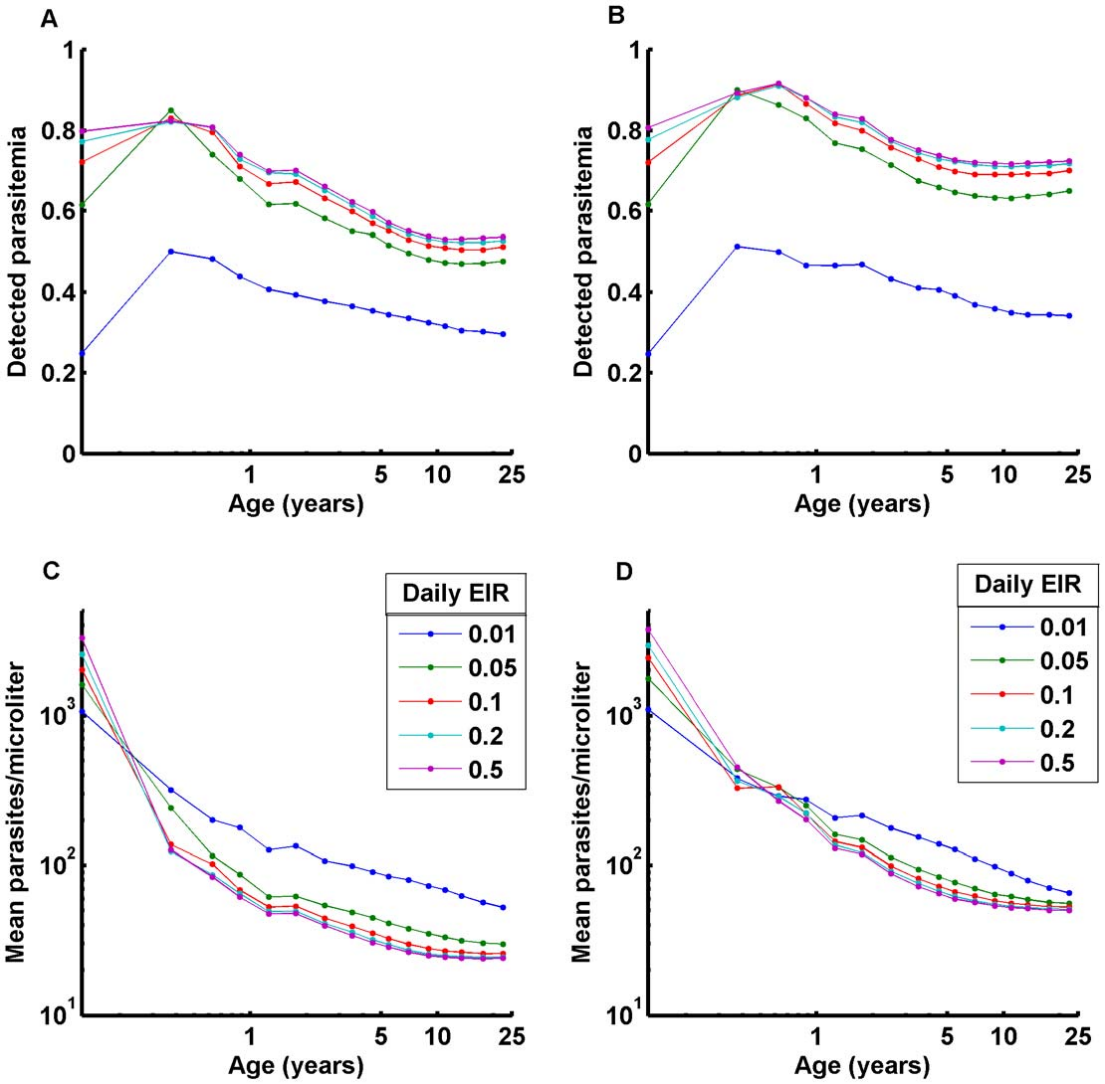
Age prevalence studies for different parasite population antigenic diversity. Age prevalence studies for A) detected parasitemia; B) geometric mean parasitemia binned by age range for a parasite population with each infection having 50 of 300 PfEMP-1 variants, 1 of 4 MSP-1 variants, and 1 of 4 sets of 5 minor epitopes; C,D) the same analysis for a parasite population with 300 PfEMP-1 variants, 10 MSP-1 variants, and 10 sets of 5 minor epitopes. Immunity takes longer to develop with a more varied parasite population.

For different transmission intensities, the detected prevalence and geometric mean parasitemia can be determined as a function of age (Figure 8). These simulations do not track maternal antibodies or different biting rates as a function of age, so these curves attain adult equilibrium levels more rapidly than are seen in data [10]. Development of immunity has been observed to be faster in adults, although they had a higher rate of clinical cases when exposed [45]. With the same parasite strain population from Figure 7A, the adult equilibria match observed patterns well: from a daily Entomological Inoculation Rate (EIR) of 0.01 to a daily EIR of 0.05, equilibrium adult-measured prevalence rises from approximately 30- to 50%, with minimal further increases at higher transmission rates. Equilibrium mean detected parasite densities are between 10 and 100 parasites/µl, with densities slightly lower in high-transmission settings. The main discrepancy with data is then the rate of transition from 1-yr-old measured prevalence and parasite densities to the 20-year-old values. This transition can be matched through various means [1], [10], but these approaches do not explicitly track antigenic history or strain structure, which is possible in this context.

In the present model, for well-mixed infections, an individual rapidly develops partial immunity to the entire antigen population at high transmission intensities, and the adult equilibrium is attained at a younger age. If it takes longer to be exposed to all antigens, the rate of approach to adult equilibrium can be slowed up to and beyond 20 years of exposure. Increasing the number of locally circulating MSP or nonspecific antigen variants can also increase the time required to develop broad immunity. Figure 8C,D repeat the age-prevalence study for a parasite population with 300 PfEMP-1 variants, 10 MSP-1 variants, 10 sets of minor epitopes, with individual infections a random selection from the local population. For a given level of transmission, it takes longer to develop immunity, and parasite rates can be higher. Thus, variation in local parasite population antigenic structure can help explain age-prevalence curves.

The detection threshold of the diagnostic will affect the field measurements: a higher detection threshold leads to lower detected parasite prevalence, but higher mean parasites per detected case. Simulation models allow specification of different diagnostics and provide the true prevalence data along with what would have been field-measured by different diagnostics. This is an important feature when comparing model results to field data or when estimating the effect of novel diagnostics on campaigns, such as mass screen and treat [46], [47].

## Discussion

The present model contains a detailed mechanistic representation of parasite and immune processes. It not only describes phenomena such as the duration of infection, but its exercising can improve the understanding of the processes that drive them. The distinctive shape of the duration distribution emerges from a balance of host immune responses and parasite antigenic switching. When the antigenic switch rate is close to the value that provides the maximum mean duration for a given set of immunological parameters, the characteristic distribution shape emerges, with a high probability density near the mean and tails characteristic both of long durations and of early clearances. The early clearances are due to the parasite’s failure to introduce a new variant before existing variants are cleared. A small number of early clearances can be tolerated by the parasite since slow switching rates result in a longer period to exhaust its antigenic repertoire. A mean duration greater than ∼200 days helps the parasite as the intense hyperimmune response that cleared the variants expressed early in the infection subsides, effectively allowing re-expression of early variants. This extends the infection, albeit with less success than during the first pass, due to the host’s immunological memory. This is a specific example of the principles of antigenic variation impacting transmission and overall pathogen fitness that are exhibited in many diseases and transmission types [48].

Mechanistic modeling of within-host parasite dynamics provides a test environment for *in silico* experiments that improve understanding of malaria infections. The peak parasite count is limited primarily by innate immune response and heterogeneity in both the level of parasitemia that provokes the response and the strength of innate immune effectors. These can explain variation in individual responses to early-stage infection. Infected human infectivity to mosquitoes cannot be explained by either a constant infectivity or a simple probability of female gametocyte presence in a bloodmeal, but requires additional factors. The first is the success at infecting the mosquito of a female gametocyte in a bloodmeal, which depends on mosquito and host-derived factors. The second is due to the host immune response, which is proposed here as being more likely the innate inflammatory response as the effect must be proportionally stronger at higher parasite counts, which is true of the innate response but not necessarily for the adaptive response. This does not rule out the effect of transmission-blocking antibodies, which have been shown to have an effect. However, within-host boosting may be weaker due to the shielding of the red-blood cell membrane [36]. These antibody levels will be important when modeling transmission-blocking vaccines, but the role of natural transmission-blocking adaptive immunity is less clear.

Accounting for the effects of parasite population strain structure on population-level prevalence and parasite counts are also important. Different degrees of explicit cross-immunity help explain field observations. Intrahost modeling can be extended along many important avenues. The first is to embed these intrahost dynamics within a population-level simulation such as [1], [3], [4], [28], [49] to observe the effects on transmission and on eradication campaign outcomes. In addition, it is necessary to incorporate age-dependence of initial response [45] to accurately determine severity of outcomes among children. Severe malaria has various causes [50], and this model will be extended to simulate infection outcomes, such as death, clinical malaria, anemia, and sequelae, as has been done particularly well by the Swiss Tropical Institute [4].

New research is continually expanding knowledge of many less well-understood areas of the immune response to malaria [51]. The detailed, flexible framework of the present model enables facile incorporation of new findings. For instance, in addition to the immune response affecting the parasite, the parasite can modulate the immune response through a variety of modalities. Infections have been found to interfere with dendritic cell maturation and to cause an increase in anergic or regulatory T-cells [52]. Active infections have been seen to damage T-cells [50], [53], an effect that may be associated with poorer-than-expected immunological memory. These mechanisms are not implemented directly in the present model, but appear indirectly in the level to which antibody capacity falls as well as the rates of its rise and decline. Drug [46], [54], [55] and potential vaccine actions [4], [56] will soon be incorporated in this model as well. It may be important to model co-infection with multiple species [57], which entails modeling of *P. vivax* and *P. malariae* at least. This could also be explored within the current framework.

Modeling a malaria infection at this level of detail can be challenging, and many prior models have illuminated the way forward. Modeling basic mechanisms constrained by specific experiments allows understanding of broader, more complicated high-level effects, such as infection durations. In contrast, models that rely heavily on non-physical parameters or fit many free parameters to observational datasets can make it difficult to generalize reliably or to gain new insight. Dramatic gains in computing power characterized by Moore’s Law currently confer on almost any research group computing capabilities adequate to model disease in much deeper levels of fidelity and detail than was feasible even a decade ago.

The present model implements a collection of experimentally-verified low-level processes that naturally reproduce complex characteristics of *P. falciparum* infections. It yields reasonably compelling explanations for subtle features of infection dynamics while maintaining clear connections to past, present, and future experiments. It also highlights several areas of study that may be critical to successful eradication.

## Materials and Methods

### Model Structure

The model implements a hybrid of discrete and continuous processes. Discrete events such as latencies in the infected hepatocyte stage, the length of the asexual cycle from merozoite invasion to schizont rupture, and gametocyte maturation are represented by timers and no state change occurs until the timer has completed. Other processes such as the decay of antibodies and the clearance of parasites are represented by continuous-time processes which are solved with a one-hour time step Euler method. All parasite quantities such as number of hepatocytes, number of merozoites, number of infected red blood cells of each antigenic variant, and gametocytes of each stage are represented as discrete integers, and the infection is not cleared until each category is reduced to zero, which allows resolution of model dynamics at sub-detection levels. Immune variables such as antibody levels for each antigen, inflammatory cytokine levels, and immunological memory are represented by continuous floating-point variables. Discrete and continuous processes work together to capture system latencies and discrete events inherent in the asexual cycle. Most model parameters have a specific physical interpretation and can be rationally constrained by defined experimental measurements.

Each infection is represented by a number of currently infected hepatocytes, the number of infected red blood cells of each PfEMP-1 variant, and the number of gametocytes in each stage of development, both male and female. The immune response is represented as the levels of non-specific inflammatory cytokines, the current antibody levels against each PfEMP-1 variant and each shared minor epitope, and the current capacity to produce antibodies of each type. For each component of the model, the sections below review what is known of the specific mechanisms, identify the relevant rates and magnitudes from the literature where available, and explain how these numbers are integrated into the model structure. Model sensitivities to these literature-derived rates are evaluated, and where specific numbers are not available for a particular mechanism and parameter, such as for the slower antigenic switching rates in the tail of the infection, the malariatherapy data or parasite prevalence data from field sites are used to constrain the parameter value through comparisons to model outputs.

The parameterization of the model follows several steps. First, several parameters are obtained from literature and used directly without fitting. These include the duration of the liver stage, the duration of the asexual stage, the number of merozoites per liver schizont, the number of merozoites per red blood cell schizont, and various other time constants governing antibody responses. The next set of parameters includes the pyrogenic threshold and the antibody stimulation threshold, which are constrained within an order of magnitude by literature estimates. That approximate magnitude is the starting point for simulations, and the results presented in this paper show the effects of varying these over the range consistent with literature estimates. The values of the effects of different immune effect strengths, which in this model are often simplified combinations of multiple mechanisms, upon parasite clearance or merozoite invasion are explored in Figure 2. Only certain ranges of combinations are consistent with observed infection behaviors, and it is within these compatible ranges, that extensive simulations were conducted and ensembles of simulated infection trajectories were generated. Malariatherapy data are then used to constrain and fit model simulation outputs to the high first parasite density peak, lower secondary peaks, and even lower peaks after 100 days, the possibility of reinfection with homologous strains, the interval between peaks, and the distribution of measured durations. The most uncertain parameter is the per-parasite antigenic switching rate. This was varied over many orders of magnitude to find the range for which distributions of infection durations were compatible with those observed in malariatherapy patients. In many cases, the desired output from the parameterization process was not a single best set of parameters, but an understanding of how parameters co-varied and affect the dynamics and true observables. This sequential process allows detailed exploration of the relative impacts of different immune and parasite parameters upon disease dynamics, as demonstrated in the Results section, and it provides a mechanistic explanation for the shape of the duration distribution.

The model is a micro-simulation of a single infection within a single individual and outputs the parasite and immune dynamics over time. Many infections are simulated in order to construct distributions of outcomes such as the measured duration of infection. This micro-simulation model can be embedded in a population-level transmission model and can also support superinfection, but these implementations are not explored here.

### Parasite Dynamics by Life Cycle Stage

The model tracks sporozoite infection of the liver, infected hepatocytes, infected red blood cells (IRBC’s) for the asexual cycle, and the production of gametocytes and their maturation. The population of each parasite stage is represented as a discrete integer, with the IRBC’s further subdivided by expressed surface antigen, and the resulting concentration per microliter is calculated from the population for use in immune response functions. *Plasmodium’s* journey begins when a female *Anopheles* mosquito with sporozoite-infected salivary glands feeds on a host [58], [59]. Sporozoites journey through the skin until reaching a capillary and entering the bloodstream, eventually arriving at the liver and infecting hepatocytes [60]. Each sporozoite-infected hepatocyte will develop into tens of thousands of merozoites [50]. The pre-patent period (time from inoculation to first detected parasites, not to emergence from liver) for malariatherapy patients infected with sporozoites ranges from 7 days up to 16 days for El Limon, 14 days for Santee-Cooper, and 28 days for McLendon [26]. Infectious bites can be modeled as a sporozoite count Gaussian distribution with a mean and standard deviation of 20 (negative counts truncated to 0), with a low probability of success per sporozoite which can depend on a variety of factors [61]. In the present model, these features correspond to an overall infectious bite success probability ***b*** [62], [63], which includes an intrinsic success of invasion and the probability of hepatocyte survival to release of merozoites. Successful sporozoites result in infected hepatocytes, which are modeled to release ***N_livermerozoites_*** = 40,000 merozoites [16] after a fixed latency ***T_hepatocyte_*** of 7 days [61].

When this micro-simulation model is embedded in a population-level agent-based simulation of the local human population, multiple homologous infectious bites in a day for a person can be replaced by the probability of at least one successfully infected hepatocyte. This gives a probability of infection with a given strain at a given time step as (1−e^−bB^) for ***b*** the probability of success per bite and ***B*** the number of bites in a given time step, after adjusting for interventions such as bednets. When embedded in a population-level simulation, a one-day time step is used between mosquito population feeding events to represent night-time biting. A one-hour time step is used to simulate the immune and parasite dynamics within each individual between mosquito-population feeding events.

Merozoites invade RBCs and begin asexual reproduction to produce more merozoites [64]. The asexual reproductive cycle for *P. falciparum* lasts approximately two days, and each IRBC produces an average of 16 merozoites [16], but that figure can climb to 32 [7]. *In vivo* growth rates before the onset of immune responses have been measured between 12 and 15 second-generation IRBCs for each original IRBC [61], [65]. In the model, the asexual cycle is tracked with a fixed latency ***T_asexual_*** = 48 hours rather than a continuous growth rate of the population, at the end of which surviving IRBCs release ***N_IRBCmerozoites_*** = 16 merozoites, and the number of resulting second-generation IRBCs depends on merozoite invasion-blocking immunity and the availability of uninfected RBCs.

IRBCs express surface proteins, such as PfEMP-1 [66], and each parasite genome has a repertoire of approximately 50 variants for PfEMP-1 encoded by the *var* gene family [67]. As these surface proteins are highly visible immunologically, antigenic variation through the *var* gene repertoire assists in immune escape, with each IRBC exhibiting mutually exclusive expression of a variant [68]. Data show switch rates of 2% per generation [69], or variable rates ranging from undetectably small to 2% [35]. Horrocks et al. found that instead of explicitly sequential transitions, expression of given variants appeared to switch on and off, with separate on and off rates for a single variant, different on/off rates across variants in the repertoire, and conserved on/off rates for a given *var* gene in genetically similar IRBCs. There appears to be deep expression-dependent epigenetic silencing of certain *var* genes [35] and a structured switching pattern which can be represented by a network of switching pathways [70]. Due to a lack of sufficient data to constrain such a switching mechanism, the present model extracts a subset of features of antigenic switching and avoids the introduction of enough free parameters for variant-specific on/off rates and variant-specific deep silencing of other variants, each of which has several orders of magnitude in experimentally-measured variability.

The variable on- and off-rates, coupled with mutually exclusive expression and the silencing mechanisms result in a time-sequence of antigenic expression looking backwards in time for each IRBC and its ancestors, with one set of dominant variants in the population replacing a prior set suppressed by immune responses. In the model, a single infection is represented with a queue of ***N_variants_*** variants, with IRBCs of a given variant probabilistically switching to new variants among the next several in the queue.

Simulations explore variations of ***n_antigenswitch_***, the number of available non-suppressed variants, and results show simulations for a value of 7. Limiting the number of available non-suppressed variants prevents the parasite from exhausting its antigenic repertoire during peak parasitemia, but excessive limitation increases the risks of early clearance upon reinfection. This implies that initially deeply-silenced or extremely low on-rate variants correspond to those later in the queue. A uniform switch rate ***K_antigen_*** is defined per IRBC rather than per variant population; hence, large populations exhibit a higher probability of introducing new variants. A switching model with on and off rates was examined by several researchers [27] who determined the basic sensitivities and some of the effects of varying these rates. As better data on constraining the distribution of on/off rates or on defining a network of structured switching become available, they will be incorporated into the model as the preferred method. The equations governing antigen switching are displayed below:
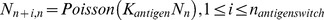
for which N_n+i,n_ is the number of IRBC’s switching from expressing antigen n to expressing antigen n+i, where N_n_ is the number of IRBC’s currently expressing antigen n. The next generation IRBC’s expressing the same antigen in the next generation N^1^_n_ is then



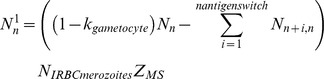
in which Z_MS_ is the merozoite success rate which is dependent on red blood cell availability and the merozoite invasion-blocking antibody response described below. The number of IRBC’s expressing an antigen in the next generation is then incremented for each of the switching IRBCs as follows:



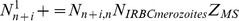
for all n, and 1≤i≤n_antigenswtich_.

The blood stage of the parasite’s life cycle exists to produce an adequacy of gametocytes in the sexual stage of *Plasmodium* [12], [15], [71], [72]. Gametocyte maturation occurs sequestered in the vasculature of internal organs rather than in peripheral circulation [73], and the process takes about 10 days for *P. falciparum* [74]. The half-life of the population of mature gametocytes is approximately 2.5 days for *P. falciparum* [75]. In the mosquito gut, gametogenesis occurs in response to the drop in temperature, the increase in pH, and the activation factor xanthurenic acid [76]. Blood meals can contain submicroscopic levels of gametocytes (<10/µl), as a bloodmeal may consist of several µl of peripheral blood) and still infect mosquitoes [77]. At submicroscopic densities, there is a positive relationship between gametocyte densities and infectivity to mosquitoes [77] which is lost at higher densities [36]. This phenomenon of infectivity of gametocytes to mosquitoes and the probability of mosquito infection [36], [38], [75], [78] can be explored through mechanistic simulations. Successful infection of a mosquito allows that mosquito to eventually infect humans following parasite development through its mosquito stages [36], [79], [80]. In the present model, a fixed fraction ***k_gametocyte_*** of IRBCs per generation produce gametocytes, which then progress through five stages of development to maturity over the course of 10 days. This framework supports stage-specific susceptibility to drugs.

### Immune System

The human immune system responds to the diverse stages of the *Plasmodium* parasite with an array of responses and effectors. Immune response to infected hepatocytes is mediated by CD8 cells [81], but NK cells are also important, as well as IL-12 and IFN-γ [82]. The model includes these pre-erythrocytic responses for vaccines.

Constructing the model for the immune response to blood stage P. falciparum requires modeling both the inflammatory innate response and the adaptive antibody-driven response. The innate immune response begins with the release of pro-inflammatory cytokines associated with a cell-mediated attack on the parasite [83]. Immune cells, such as macrophages, release TNF-α, IFN-γ, and NO [50]. In *P. chabaudi* infections in mice, NK cell-released IFN-γ and TNF-α are important to the initial control of the infection [84], [85], and disruption of the inflammatory response is dangerous to the host. Hemozoin provokes an additional innate immune response upon schizont rupture [86], which is partly responsible for the waves of inflammation and fever associated with the periodic large-scale rupture of schizonts. TNF-α and IFN-γ appear within 12 hours, stimulated by schizont ruptures and intact IRBCs once they are beyond the ring stage and express surface proteins [87]. IFN-γ appears rapidly due to NK cells stimulated by infected erythrocytes, with optimal production at ratios between 1–10∶1 IRBC:PBMC [88]. On average, the inflammatory response to primary infection tends to become important as concentrations surpass 100 IRBC/1000 PMBC or 100 IRBC/µl, with stronger responses correlated with an increase in parasite densities towards 10,000 IRBC/µl, and this is the range explored in model simulations.

The pro-inflammatory cytokine cascade activates fever and cell-mediated inflammatory responses. Fever is associated with TNF-α [89], and high levels of TNF-α are associated with clinical symptoms, with increased transcription of TNF-α is associated with risk of cerebral malaria [83]. Clinical symptoms tend to be inversely related to adaptive antibody responses, with clinical disease associated with expression of antigenic variants for which patients lacked pre-existing antibody responses [90].

The model includes the effect of the development of the antibody response shifting the immune response away from inflammatory cytokines. Specific antibody responses to GPI in MSP-1 and MSP-2 help limit the inflammatory response [91]. Clinical disease associated with the inflammatory response tends to result from novel antigens to which an antibody response has not yet developed. This helps explain differences among primary and secondary infections and the differences in time scales for development of clinical and parasitological immunities [1], [41]. The inflammatory response is regulated by anti-inflammatory cytokines, such as IL-10 and TGF-β. Low levels of IL-10 are present in patients who experience severe malarial anemia [92], and IL-10 appears as an anti-inflammatory component of acquired immune processes [93]. In mice, early appearance of TGF-β suppresses the inflammatory response and is harmful, while later appearance helps prevent severe malaria [83], [94], [95].

The present model represents the above features, time constants, and parasite density thresholds for the innate inflammatory response as follows: a variable ***Y_innate_*** representing pro-inflammatory cytokines, such as TNF-α and IFN-γ, is stimulated by rupture of IRBCs and by the presence of live IRBCs expressing variants for which there is no antibody response. The degree of stimulation has a functional form that depends on a pyrogenic threshold ***X_50,innate_***, a common concept in intrahost models [6], [10], [27]. Stimulation of the innate response is reduced as the level of antibody to that variant, ***Y_antibody,i_***, increases. Heterogeneity observed in the strength of an individual’s NK-IFN-γ response [88]can be represented by varying the threshold for stimulation and the efficacy of the response. With ***X_i_*** the concentration of IRBCs of variant i, the equations for the innate immune response are
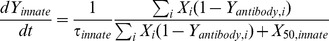



An additional variable, ***Y_fever_***, represents fever, which is a scalar function of the level of pro-inflammatory cytokines ***Y_innate_***, scaled to the appropriate temperature range in C.
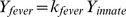



Cytokines respond to stimulation by live cells and decay with a time constant of τ_innate_ = 12 hours, but are modeled as responding immediately to the large-scale rupture of schizonts with a step-function increase.
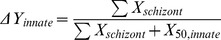



The innate inflammatory response gives way to an adaptive antibody response, characterized by growth in antigen-specific antibody production and concentrations [52], [96], [97]. In mice without B-cells, acute infections of *P. chaubaudi* are controlled but not cleared [83]. This shows that while the inflammatory response is necessary for rapid control, the adaptive response is needed for final clearance. In mouse models, increased levels of anti-inflammatory cytokines are associated with parasite clearance by antibodies [98]. As new antigenic variants are expressed, the repertoire of effective B-cells expands and better protects the individual against future infections [90], [96].

The model includes antibody responses to single PfEMP-1 variants, including the time course of activation, rapid proliferation of antibody response during hyperimmunity, slow loss of hyperimmunity, and residual immunological memory. Specific antibody levels tend to rise within the first week after clinical diagnosis [96], setting the time constant between 4–7 days. Antibody response to different merozoite-associated proteins has also been observed [44], [99], and antibodies to gametes can interrupt fertilization in the mosquito gut [72]. Human heterogeneity characterizes both innate and adaptive immune responses. HIV-1 co-infection, for example, impairs an effective antibody response to malaria [100]. The antibody response is separated into variables for antibody production capacity ***Y_capacity,i_*** and antibody level ***Y_antibody,i_*** for each antigen.

In the model, if any antigen is present, there is growth in ***Y_capacity,i_*** towards a maximum value of 1 for response to that antigen with a growth rate dependent on the concentration of the antigen. Values of ***Y_capacity,i_*** above 0.4 correspond to hyperimmunity, and ***Y_capacity,i_*** then grows towards 1 with a time constant of ***τ_hyperimmunity_*** = 3 days, regardless of antigen concentrations. This corresponds to the rapid (∼10 hour doubling time) proliferation of antibody-producing cells during a hyperimmune response. Many models for malaria infection incorporate a threshold for the antibody response [6], [13], [27], and the density of each antigen modulates the growth in ***Y_capacity,i_*** until hyperimmunity is attained. A maximum growth rate of 0.1, representing an initial growth rate of 4 days until hyperimmunity, is multiplied by the antigenic factor
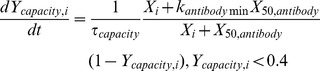


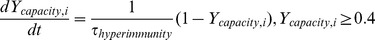
in which ***X_i_*** is the density of parasites expressing that antigen, and ***X_50,antibody_*** is analogous to the adaptive immune threshold in other models, but here is the density at which growth in ***Y_capacity,i_*** is half its maximum. ***k_antibodymin_*** is the minimum growth rate at low antigen concentration, occasionally relevant upon reinfection in certain parameter regimes. This ***Y_capacity,i_*** represents both the helper T-cell and antibody-releasing B-cell repertoires specific to that antigen. If ***Y_capacity,i_*** >0.3, antibody is released in the presence of antigen, and antibody levels ***Y_antibody,i_*** rapidly approach ***Y_capacity,i_*** with a time constant ***τ_abprod_*** = 6 hours if X_i_ >0.

Antibody levels are multiplied by a kill rate. The higher values of antibody during hyperimmunity correspond to both higher antibody densities and enhanced antibody affinity for antigen after somatic hypermutation resulting in affinity maturation. The resulting antibody levels during the initial infection resemble the growth in specific antibody levels measured in clinical patients [96].

In addition to antibodies to different PfEMP-1 major variants, the model includes antibodies to minor epitopes on IRBC surfaces [33]. In the present model, each IRBC has a major PfEMP-1 variant and 1 minor epitope variant. The number of minor epitope variants available per clone is unknown, but several options were explored, with results shown for 5 minor epitopes per clone. Since the minor epitopes are less immunogenic, the growth rate of this antibody response is reduced by a factor ***k_minormod_*** = 0.5.

The merozoite antibody response is a single ***Y_capacity,MSP_*** and an antibody level ***Y_antibody,MSP_*** which gathers the responses to all important merozoite proteins, such as MSP-1 and AMA-1. Antibody responses have been measured for several merozoite proteins [44], [99], [101], and as antibody levels increased, merozoite invasion was inhibited *in vitro* by 50–75% [101]. A recent study found protection associated with increasing levels of MSP-1 antibodies, but not MSP-2 [102]. The ***Y_capacity,i_*** for merozoite proteins is modeled differently than for other antigens, since the merozoites are visible extremely briefly as they transit between RBCs. Every two days when the schizonts rupture, ***Y_capacity,i_*** increases by a constant ***K_MSP_*** multiplied by the density sigmoid from above, with the number of released merozoites as P.
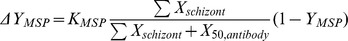



This ***K_MSP_*** is unknown, and the effects of its variation are explored. Once ***Y_capacity,i_*** >0.4, hyperimmunity begins as with dynamics as for the other antibodies.

Immunological memory is an important component of the human immune system, but many questions remain about how it operates against *Plasmodium* [51]. One study showed that counts of memory B-cells were much lower for tested malaria antigens than for tetanus, and some subjects had antibody but no detected memory cells [103]. However, a report from Madagascar found sustained defenses years after the interruption of most malaria transmission [104].

The parameter ***Y_memory_*** defines the strength of immunological memory. In the absence of antigen, if ***Y_capacity,i_*** > ***Y_memory_***, ***Y_capacity,i_*** decays towards ***Y_memory_*** with a time constant ***τ_capdecay_*** corresponding to loss of hyperimmunity approximately 120 days after disappearance of antigen. Antibody levels ***Y_antibody,i_*** decay towards zero with a time constant ***τ_abdecay_*** of 20 days. Immunological memory is modeled as ***Y_capacity,i_*** staying at ***Y_memory_*** in the absence of antigen, which is closer to a return to hyperimmunity than 0. The growth rate upon reinfection is as before, but starting at ***Y_capacity,i_*** = ***Y_memory_*** and with suppressed IRBC counts compared to primary infection. Once ***Y_capacity,i_*** = 0.4, hyperimmunity returns with a rapid proliferation of antigen-specific B-cells and T-cells. These different thresholds represent fundamental details of the antibody response, such as the shortened response time upon reinfection, the rapid growth in the strength of the antibody response during hyperimmunity, and its decay over intervals of several months. These threshold values are fixed in the present model, but the growth rates can be varied to change the effect. Future versions of the model may include a gradual decay of ***Y_capacity,i_*** from ***Y_memory_*** towards 0 in the absence of stimulation with a 2–10 year time constant.

### Parasite-Immune System Interaction

This section describes the model component for the effects of immune responses on different stages of the parasite’s life cycle. The model incorporates an antibody response that limits the success of merozoite invasion, an innate inflammatory response that both assists in clearing asexual-stage parasites and limits gametocyte success in the mosquito, and an antibody response to PfEMP-1 and minor epitopes that clears asexual-stage IRBCs. Antibodies to merozoite surface proteins have been found to inhibit merozoite invasion [101] with invasion success reduced to 25–50% compared to controls, at high antibody levels for two different strains. In the model, merozoite invasion is blocked by the factor ***C_merozoite_Y_antibody,MSP_***, such that Z_MS_ = (1 - ***C_merozoite_Y_antibody,MSP_***). The kill rate for IRBC’s due to the inflammatory immune response is due to TNF-α and other components of the inflammatory wave that can damage asexual stages as mentioned earlier [36], [105]. The present model groups all these effects into the single inflammatory kill rate parameter, ***C_innate_***, and the resulting kill rate is set to be a sigmoidal function of ***Y_fever_*** to limit clearance at low levels and saturate at high levels of fever. The saturation in the effect of fever allows parasite growth to outrace the limiting effect in certain parameter regimes.
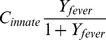



The model also includes clearance of IRBC’s mediated by the adaptive antibody response to specific antigens. Knobless IRBCs experience increased clearance by the spleen [106], and antibody interference with adhesion mediated by surface proteins in the knobs could increase IRBC clearance via this modality. Antibody-MN interaction inhibits IRBC development and kills the parasite [107]. Other possible mechanisms include antibody mediated activation of innate immune effectors. These antibody-dependent effects are included in the kill rate. Although due to separate antibody populations, all responses to a specific antigen are grouped into a single kill rate ***C_antibody_*** in the model. In future versions, these effects could be separated as the mechanisms become better understood. The kill rates ***C_innate_*** and ***C_antibody_*** **each** include the collective effects of several mechanisms, and thus, are not well-constrained by direct data. These two parameters can be varied, and the observed effects on peak parasite count and durations provide constraints on their possible values when compared to malariatherapy data. The parameter ***c_minormod_*** modulates ***C_antibody_*** for minor epitopes, and variation in this parameter from 0 (no effect) to 1 (same strength and effect as antibodies to PfEMP-1) is explored. The probability of destruction for a single IRBC of a given variant is then calculated, with binomial distribution of the number of cleared IRBCs of that variant
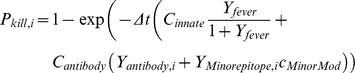



The present model supports a detailed implementation of superinfection. An individual can maintain multiple infections, all interacting with the same immune system. Identical antigens from different infections are summed across infections for immune stimulation, with the immune system responding to the total specific antigen, regardless of its parent infection.

### Summary of Model Equations

There is a 1-day time step for the interaction of an individual with the vector population, and over a given night of vector biting, an individual will receive B bites, each with a probability b of success. The probability of adding a new infection that time step is then
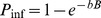



A random draw for that individual determines whether a new infection on that time step will occur. If so, another infection is added to that individual, and a new hepatocyte timer ***t_hep_*** is started and initialized at ***T_hepatocyte_*** = 7 days.
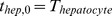



For the disease dynamics within the individual, a 1-hour time step Δt is used, and ***t_hep_*** counts down to start of blood-stage infection.
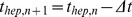
When ***t_hep_***≤0, the liver schizonts rupture and the blood stage begins. The antigenic repertoire for the blood stage is set up with 50 random antigens arranged in an array, each with one out of 5 minor epitopes. The asexual stage timer ***t_asexual_*** is initialized to ***T_asexual_*** = 48 hours.



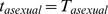



The ***N_livermerozoites_*** = 40000 merozoites from the liver create the initial set of IRBC’s that make up the blood stage. These are divided equally among the first five antigens in the array of 50.

For i = 1∶5, 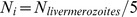


While the asexual timer decreases towards zero, both inflammatory and antibody immune responses are stimulated and IRBCs are cleared, with dynamics governed by the equations below. Note that ***X_i_*** is the concentration of IRBCs of a given antigen, so that ***X_i_*** = ***N_i_***/(number of microliters of blood).
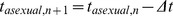


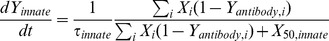


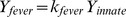



For i = 1 to 50, if ***X_i_*** >0,
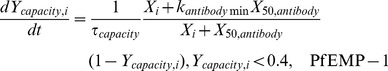


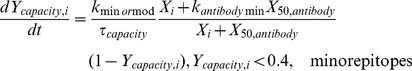


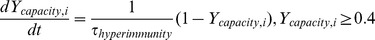





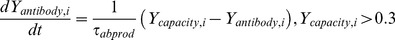


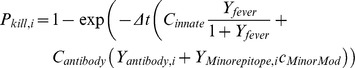





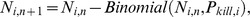
the n and n+1 indicate successive time steps

For i = 1 to 50, if ***X_i_***_ = _0,
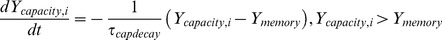


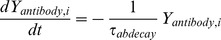



Once the timer ***t_asexual_*** reaches zero, a series of discrete steps occur and these dynamics are not continuous like the above equations. The schizonts rupture, there is additional transient inflammatory stimulation, merozoite-specific immunity develops, antigenic switching occurs, and immature gametocytes advance a stage.
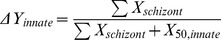


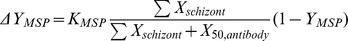



Calculate the number of IRBCs of antigen n switching to express antigen n+i:
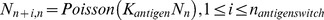



Calculate the number of IRBCs of antigen n created by previous generation expressing antigen n:
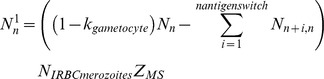



For all n, i = 1 to n_antigenswtich_, add in the switching IRBCs.
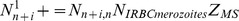
In which



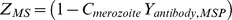



Finally, gametocytes are produced and mature through stages,
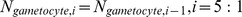


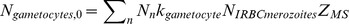
and the asexual timer is reset for the next generation.



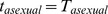



A summary of the computational algorithm per time step is provided in Supporting Information S1. Biological input parameters are listed in Table S1, and the model variables are found in Table S2.

## Supporting Information

Table S1 **Biological input parameters of the model.**
(DOC)

Table S2 **Key model variables.**
(DOCX)

Supporting Information S1 **Summary of the computational algorithm per time step.**
(DOC)
