# Supplementary material for: P. falciparum Infection Durations and Infectiousness Are Shaped by Antigenic Variation and Innate and Adaptive Host Immunity in a Mathematical Model
Source: PLoS One. 2012 Sep 19;7(9):e44950. doi: 10.1371/journal.pone.0044950 (PMC3446976; doi:10.1371/journal.pone.0044950)
Supplement: Table S1 — Biological input parameters of the model. (DOC) [file pone.0044950.s001.doc]

**Table S1:** Summary of biological input parameters

| Immunological |  |  |  |
| --- | --- | --- | --- |
| X_50,innate_ | Parasitemia at which innate immune response rate is half of maximum | C_innate_ | Rate at which innate immune system kills IRBCs |
| X_50,capacity_ | Parasitemia at which antibody immune response rate is half of maximum | C_antibody_ | Rate at which specific antibodies kill IRBCs |
| τ_innate_ | Inverse of maximum innate immune response rate | C_minormod_ | Modification of antibody kill rate for minor epitopes |
| τ_capacity_ | Inverse of maximum antibody response rate | C_merozoite_ | Maximum blocking of merozoite invasion by MSP antibodies |
| τ_hyperimmunity_ | Inverse of rate of development of hyperimmunity | K_MSP_ | Per cycle growth rate of MSP antibody response |
| k_antibodymin_ | Minimum antibody response to antigen | Y_memory_ | Immunological memory level |
| k_minormod_ | Modification of antibody response rate for minor epitopes | T_hyperimmunity_ | Duration of hyperimmunity after removal of antigen |
| k_gametocyte_ | Base success rate per female gametocyte in a mosquito | C_50,gametocyte_ | Cytokine level Y_innate_ at which gametocyte inactivation is 0.5 |
| Parasitological |  |  |  |
| N_livermerozoites_ | Merozoites per hepatic schizont | K_antigen_ | PfEMP-1 antigenic switch rate |
| N_IRBCmerozoites_ | Merozoites per IRBC schizont | k_gametocyte_ | Gametocyte production rate |
| T_hepatocyte_ | Duration of liver-stage | T_gametocyte_ | Duration of gametocyte maturation |
| T_asexual_ | Duration of asexual cycle | τ_gametocyte_ | Decay of mature gametocytes |
| N_variants_ | Number of PfEMP-1 variants per clone | n_antigenswitch_ | Number of non-suppressed available variants per IRBC |
| N_minorepitopes_ | Minor epitopes per clone |  |  |

*k_fever_ multiples Y_innate_ to get Y_fever_, which is multiplied by C_innate_, so k_fever_ and Y_fever_ are mathematically redundant and just present for scaling of the fever output)
