# Supplementary material for: P. falciparum Infection Durations and Infectiousness Are Shaped by Antigenic Variation and Innate and Adaptive Host Immunity in a Mathematical Model
Source: PLoS One. 2012 Sep 19;7(9):e44950. doi: 10.1371/journal.pone.0044950 (PMC3446976; doi:10.1371/journal.pone.0044950)
Supplement: Table S2 — Key model variables. (DOCX) [file pone.0044950.s002.docx]

**Table S2:** Summary of variables in the model

| N_n_ | Number of IRBC’s expressing antigen n |
| --- | --- |
| N_n,n+i_ | Number of IRBC’s expressing antigen n whose merozoites will create IRBC’s in the next generation expressing antigen n+i |
| N^1^_n_ | Number of IRBC’s in the new asexual cycle expressing antigen n |
| X_i_ | Concentration per microliter of parasites expressing antigen i |
| Y_innate_ | Level of pro-inflammatory cytokines |
| Y_fever_ | Level of fever due to pro-inflammatory cytokines |
| Y_antibody,i_ | Level of antibodies specific to antigen i |
| Y_capacity,i_ | Variable representing changing capacity to produce antibodies specific to antigen i |
