# Supplementary material for: P. falciparum Infection Durations and Infectiousness Are Shaped by Antigenic Variation and Innate and Adaptive Host Immunity in a Mathematical Model
Source: PLoS One. 2012 Sep 19;7(9):e44950. doi: 10.1371/journal.pone.0044950 (PMC3446976; doi:10.1371/journal.pone.0044950)
Supplement: Supporting Information S1 — Summary of the computational algorithm per time step. (DOC) [file pone.0044950.s003.doc]

Supporting Information S1

Computational Algorithm for a Time Step

In this section we describe the computational algorithm for a time step of the simulation of all current infections and the immune system. The algorithm contains a mixture of continuous processes and discrete events.

1. Move each infection through a time step
   1. Process hepatocytes
      1. Increment latency timer (arithmetic)
      2. If latency timer is greater than the latent period, release merozoites (discrete event)
   2. Process asexual stage
      1. Increment asexual cycle timer (arithmetic)
      2. If asexual cycle timer is complete, calculate the effects of schizont rupture (discrete event)
         1. Calculation of number of rupturing schizonts of each variant switching to each new possible variant (Poisson[***K_antigen_***X_j_])
         2. Calculation of number of next generation IRBCs per variant
            1. rupturing schizonts for each variant multiplied by merozoites per schizont multiplied by merozoite survival
         3. Some schizonts’ merozoites chosen to produce gametocytes in next generation IRBCs
      3. Summation of antigenic presence for effect on immune system (arithmetic)
      4. Clearance of IRBCs due to cumulative kill rate of innate and adapted immune response (conversion of continuous rate to discrete probability of clearance during time step)
         1. Calculate probability of each IRBC of a specific variant being cleared
            1. P_kill_ = 1-exp(-kill rate * Δt)
         2. Number killed is drawn from a binomial distribution
   3. Process immature gametocytes
      1. Decrement gametocyte stage timer
      2. If timer expires, move all gametocytes forward a stage (discrete)
      3. Death of immature gametocytes (continuous rate converted to probability of time step survival)
2. Move immune system forward a time step
   1. Inflammatory immune response
      1. Calculate total cytokine stimulation due to surface antigens from all infections, adjusted for current antibody levels (arithmetic)
      2. Change in level of inflammatory cytokines based on rate of stimulation and decay rate (continuous; Euler method)
      3. Transient jump in cytokines due to schizont ruptures during time step (discrete event)
      4. Calculate fever based on inflammatory cytokine concentration (arithmetic)
   2. Adaptive immune response
      1. Calculate stimulation for each specific antigen, summed over all IRBCs in all infections (arithmetic)
      2. Increment antibody capacity for each specific antigen based on stimulation (continuous; Euler method)
      3. Release of antibodies if antigen present is based on current antibody capacity (continuous; Euler method)
      4. Decay of antibody if no antigen is present (continuous, Euler method)
      5. Decay of antibody capacity toward memory level (continuous, Euler method)
3. Gather mature gametocytes from all infections and calculate net infectiousness due to total number of mature gametocytes, letting X_gamF_ be the total number of mature female gametocytes in a 2 µL bloodmeal:
   1. The probability of infecting a mosquito is calculated as 1-exp(-X_gamF_ *k_gametocyte_ *(1- Y_innate_/(Y_innate_+C_50,gametocyte_))).
4. Death of mature gametocytes (continuous decay rate converted to probability of death during time step)
5. Check for clearance of infections and remove cleared infections from queue
6. Check for death of the individual
